# Progress in Gynæcology

**Published:** 1901-06-08

**Authors:** 


### Progress in Gynæcology.

**Amenorrhœa.**—Arthur E. Giles[1] formulates the following general principles as a guide in giving a prognosis when consulted in any given case on account of the non-establishment of menstruation. The two points on which we shall most probably be asked to express an opinion are: First, the likelihood of menstruation coming on; second, the likelihood of child-bearing in case of marriage. With regard to the likelihood of menstruation coming on, our forecast will depend in the first place on the patient's age. Even without examination we can give a good prognosis in the case of a girl of sixteen or seventeen; at eighteen or nineteen the prognosis must be more guarded; and after the age of twenty no opinion should be expressed without making an examination. In the second place we should be guided by the general health of the patient. If she be suffering from any constitutional condition that may cause amenorrhœa, we shall be able to say (with the proviso of age just mentioned) that menstruation will probably begin when the general health has improved. Supposing the general condition to be good and the patient twenty years of age or older, an examination should be made, and if no abnormality can be found or the uterus is only slightly under the normal size, the prognosis is not unfavourable; if, however, the uterus is rudimentary, the probability is against the establishment of menstruation.

With regard to the forecast as to childbearing, the first thing necessary will be to examine the pelvic organs. If these are found to be normal, we can say that if the patient be eighteen the prognosis is still fairly good; if she is nineteen and has not menstruated she is more likely to be relatively, though not absolutely sterile. If she is twenty or over, the likelihood of childbearing diminishes rapidly in proportion to the age; and if she is 25 or older she will almost certainly be sterile. J. B. Hellier[2] records three interesting cases of amenorrhœa due to anatomical defects. In two of the cases the uterus was rudimentary, bifid, and unsymmetrical; in the third case, on examination, an imperforate hymen with hæmatocolpos and hæmatometra were found; although there had been no history of menstrual molimina, and only a little abdominal pain. Hellier points out that the most serious danger in these cases arises when hæmato-salpinx is present as well as hæmatocolpos and hæmatometra. The danger is twofold. Firstly, the thinned and distended oviduct has often contracted adhesions, and may rupture when the great mass of accumulation is liberated. Secondly, the hæmato-salpinx is apt to become septic. This is due, he thinks, to the fact that it does not contract and empty itself as a distended uterus will. Hence he maintains that if hæmatosalpinx is present it is advisable to perform abdominal section and remove the tubes.

**The Treatment of Inflammatory Affections of the Uterine Appendages.**—During the last few years there has been an increasing tendency to conservative methods in the treatment of inflammatory affections of the uterine appendages. J. A. C. Kynoch[3] says conservative methods should be practised whenever practicable, and that such a line of treatment is correct has been abundantly proved by the frequent occurrence of pregnancy after the unilateral removal of diseased appendages. He quotes a number of operators in support of his views. W. M. Polk,[4] discussing the subject, says that the importance of such work is self-evident, but surgeons have been slow to adopt such methods, because they were loth to leave behind in a tissue like the peritoneum any disease which might perhaps jeopardise the life of the patient. Now that the propriety of conservative surgery in this field has been demonstrated, the importance of such work increases with the extent of the new field opened up. W. L. Burrage[5] records the immediate and remote results in 100 cases. A symptomatic cure had been noted in 73 out of the 100 cases, and 34·5 per cent. of the women who had previously borne children had become pregnant after the operation. When the tubes were open at the time of operation pregnancy was common, even though only a small fragment of ovary was allowed to remain; but in no instance had pregnancy occurred after resection of a closed tube, both tubes being closed at the time of operation.

Fourness Barrington,[6] while admitting that in some cases removal of the appendages is absolutely necessary, in others, even if there be marked displacement of the uterus and ovaries and fixity of these organs, it is by no means necessary to sacrifice them, for an exploratory operation with liberation of organs from adhesions, and their replacement may be good surgery, while a radical operation would not. He strongly maintains that preliminary dilatation of the cervix, curetting, and gauze drainage of the uterus should be carried out in every inflammatory case immediately before abdominal section. He says that the surgeon who removes the appendages for advanced inflammatory disease, and leaves the uterus still the primary focus in the pelvis, is not founding his work on a sound pathological basis, and it at best ranks as indifferent surgery. C. S. Hawkes[7] emphasises the fact that cases of gonorrhœal tubal disease sometimes improve under treatment by mercury and iodides without one being able to trace any specific infection. He quotes cases of his own when marked improvement followed the use of these drugs, and he would limit operative interference to cases where gross lesions of the tubes and ovaries are present.

**Extrauterine Pregnancy.**—J. H. J. Upham[8] reports an interesting case of extrauterine pregnancy, in which the diagnosis was made previous to rupture, the abdomen opened and the pregnant tube removed. The patient, aged 20, nullipara, was four days overdue in her menstruation, when rather severe uterine hæmorrhage came on, and lasted a week. Two days later she was seized with cramp-like abdominal pains, when a decidual membrane was discharged. On examination the cervix was found to be softened, the os patulous, the uterus very slightly enlarged, in good position, and freely movable. To the left side and a little anteriorly was a mass distinct from the uterus, movable, elastic to the touch, and distinctly tubular in shape, ovary normal, as were also the appendages on the right side. From the absence of constitutional symptoms, the lack of evidence, or history of gonorrhœal or other infection, together with the significant history, the diagnosis of an unruptured left tubal pregnancy was made, and operation advised. The abdomen was opened and the left tube and ovary removed without difficulty. The removed tube was eight c.m. in length and two c.m. in diameter; the greatest distension was in the ampulla, and here the peritoneum was drawn tightly over the thinned coats of the tube stretched almost to rupture. The contents of the tube proved to be laminated clot; an accessory ostium led directly into this sac, and the writer could find no direct connection with the normal lumen of the tube; the fertilised ovum must therefore probably have entered the secondary ostium and started developing in this abnormal cavity. A. T. Bristow[9] draws attention to the difficulty sometimes met with in distinguishing between appendicitis and a right-sided tubal pregnancy. He believes that quite a large number of cases of tubal pregnancy are mistaken for appendicitis; and the principal point on which he depends in making a diagnosis is that in appendicitis there is marked spasm of the right rectus muscle, while in tubal cases (although there may be tenderness and some resistance in the abdominal wall) the intense spasm of the rectus which is present in appendicitis, is absent.

**Intra-peritoneal Rupture of Ovarian Cysts.**—F. W. N. Haultain[10] reports six cases of intra-peritoneal rupture of ovarian cysts, and he draws attention to the fact that in many cases there is a curious absence of symptoms accompanying this complication; this is due doubtless to the innocuous contents of the cysts and to their slow escape, a sudden gush being prevented by intra-abdominal pressure, the peritoneal cavity being merely potential. The causes of rupture are numerous, such as violence, morbid processes in the cyst wall, intrascystic hæmorrhage, and nutritive disturbances of the wall, the result of torsion of the pedicle. The commonest variety of cyst to rupture spontaneously is probably that with gelatinous contents, the cyst wall becoming secondarily softened. The contents of the cyst though absorbable, may be irritative and thus cause peritonitis, with adhesions so widespread as to prove an almost insuperable barrier to its removal. In some cases no absorption takes place, the rent in the cyst wall remains patent, and active secretion continues, the abdomen steadily distending with intra-peritoneal fluid. The treatment of ruptured cysts consists in their removal as soon as possible. A certain proportion of cases after rupture seem never to re-distend, and a spontaneous cure results, but as delay might allow strong and widespread adhesions to form early operation is advisable. Anchê and Chavannaz,[11] writing on the lesions produced by the intra-peritoneal rupture of ovarian cysts point out that the fluid may disseminate elements of new growth formation throughout the peritoneum, it may give rise to peritonitis by distributing organisms throughout the cavity, or it may give rise to certain secondary visceral changes; the liver and kidney on account of their excretory function being the organs affected. To study the pathological changes in these organs, cyst contents were injected into the peritoneal cavity of rabbits. Within a variable time after absorption there were found parenchymatous changes (necrosis, cloudy swelling, hyaline degeneration) and sclerosis of the liver. Similar changes were present in the kidney. These changes the writers maintain are not due to any microbic infection, but to a certain toxic influence possessed by the cyst contents.

**The Treatment of Fibromyomata of the Uterus.**—In no branch of surgery have greater strides taken place during the last few years than in the treatment of fibromyomata of the uterus; the mortality of abdominal hysterectomy having been reduced during the last fifteen years from 60 to 5 per cent. E. Malins[12] discussing the indications for operative treatment in these cases, deals with them under the following headings:—(1) Social position. This is often an important consideration, operation being more urgent in a patient of the working class, who cannot afford to lead the life of a semi-invalid. (2) Excessive and persistent hæmorrhage. This undoubtedly is a ground for interference, as in cases of this kind we know of no medical treatment that admits of a safe prediction as to results. Although ergot has been lauded as a standard prop by some, it is at the best very unreliable. (3) Pain and discomfort due to pressure symptoms sometimes entail a consideration of interference. This is most frequent when the tumour is in the pelvis, where it encroaches upon neighbouring viscera or nerves, or distends the uterine walls, giving rise to suffering of a severe kind. (4) Rapid growth of the tumour. Here it is often difficult to form a decisive opinion, owing to the fact that these fibroids sometimes grow rapidly for a time, and then stop without any apparent cause. When, however, the rate of growth is unquestionable, and particularly if there are signs of associated complications, operation is imperative. Without by any means advocating operation in all cases of uterine fibroids, Dr. Malins says that the trend of modern surgery is to operate before the more severe symptoms appear, while the tumour is comparatively small, and the patient's general health not undermined. He recommends the abdominal operation for all tumours of any size, and leaves the cervix whenever possible. He thinks the Trendelenburg position a very desirable adjunct if it can be obtained, as it adds greatly to the advantage of sight and facility of manipulation. After ligaturing and dividing the broad ligament on both sides, the cervix is cut through from above downwards by a V-shaped incision at the front and back forming two flaps; these are brought together by silk sutures, and the peritoneum brought over the stump.

**Malignant Disease of the Uterus.**—The cases of malignant disease of the uterus cured by surgery are only a fraction of the number that run their course unaffected by treatment, and this in the majority of cases is due to the fact that the disease is not recognised until it has advanced by direct extension into parts adjoining the uterus. Richelot[13] writing on this subject says he believes that cancer is primarily an atypical epithelial proliferation, which, due to some nutritive trouble of the organism, invades adjacent tissues, and finally forms metastatic tumours in different parts of the body. Uterine cancer exists in two stages: in the first it is a local disease, limited to the uterine tissues; later it invades the neighbouring structures. The relative duration of the two periods is variable, and depends partly on the type of growth, and partly on unknown conditions of the organism. The object of all surgical treatment is to interfere in the first stage whilst the growth is limited to the uterus. A. H. N. Lewers[14] draws attention to the fact that patients suffering from cancer of the uterus may, and generally do for a relatively long period, look quite well. They may be well nourished, and as regards the aspect of the face, may appear to be in perfect health; the only symptom present to call attention to the existence of the disease during this early stage being irregular hæmorrhage. There is a good deal of difference of opinion at the present time as to the relative merits of abdominal and vaginal hysterectomy in cancer of the uterus. Mangiagalli[15] favours the vaginal route. He says that recurrence after operation is most commonly local in the vaginal sear, and this is not avoided in any way by abdominal hysterectomy. H. J. Boldt[16] considers that only in exceptional cases should abdominal hysterectomy for cancer be performed, until we are in possession of facts proving its superiority over vaginal hysterectomy in ultimate results.

[1] Clin. Jour., Jan. 30, 1901. [2] Quarterly Med. Jour., Feb., 1901. [3] Scottish Med. Jour., Feb. 1901. [4] Med. Rec., Feb. 16, 1901. [5] Med. Rec., Feb. 16, 1901. [6] Australasian Med. Gaz., Nov. 20, 1900. [7] Australasian Med. Gaz., Dec. 20, 1900. [8] The Columbus Med. Jour., Dec. 1900. [9] Brooklyn Med. Jour., Nov., 1900. [10] Edin. Med. Jour., Feb. 1901. [11] Brit. Med. Jour., Mar. 2, 1901. [12] Lancet, Dec. 8, 1900. [13] Med. Chron, Dec., 1900. [14] Lancet, Jan. 5, 1901. [15] Med. Chron., Dec., 1900. [16] Med. Rec., Dec. 8, 1900.

